# Identification and characterisation of capidermicin, a novel bacteriocin produced by *Staphylococcus capitis*

**DOI:** 10.1371/journal.pone.0223541

**Published:** 2019-10-16

**Authors:** David Lynch, Paula M. O’Connor, Paul D. Cotter, Colin Hill, Des Field, Máire Begley

**Affiliations:** 1 Department of Biological Sciences, Cork Institute of Technology, Cork, Ireland; 2 Teagasc Food Research Centre, Moorepark, Fermoy, Cork, Ireland; 3 APC Microbiome Ireland, University College Cork, Cork, Ireland; 4 School of Microbiology, University College Cork, Cork, Ireland; Department of Health, Hong Kong, HONG KONG

## Abstract

One hundred human-derived coagulase negative staphylococci (CoNS) were screened for antimicrobial activity using agar-based deferred antagonism assays with a range of indicator bacteria. Based on the findings of the screen and subsequent well assays with cell free supernatants and whole cell extracts, one strain, designated CIT060, was selected for further investigation. It was identified as *Staphylococcus capitis* and herein we describe the purification and characterisation of the novel bacteriocin that the strain produces. This bacteriocin which we have named capidermicin was extracted from the cell-free supernatant of *S*. *capitis* CIT060 and purified to homogeneity using reversed-phase high performance liquid chromatography (RP-HPLC). Matrix-assisted laser desorption/ionization time-of-flight (MALDI-TOF) mass spectrometric (MS) analysis revealed that the capidermicin peptide has a mass of 5,464 Da. Minimal inhibitory concentration (MIC) experiments showed that capidermicin was active in the micro-molar range against all the Gram-positive bacteria that were tested. Antimicrobial activity was retained over a range of pHs (2–11) and temperatures (10–121°C x 15 mins). The draft genome sequence of *S*. *capitis* CIT060 was determined and the genes predicted to be involved in the biosynthesis of capidermicin were identified. These genes included the predicted capidermicin precursor gene, and genes that are predicted to encode a membrane transporter, an immunity protein and a transcriptional regulator. Homology searches suggest that capidermicin is a novel member of the family of class II leaderless bacteriocins.

## Introduction

The rise and spread of multidrug-resistant bacterial pathogens, coupled with a diminishing repertoire of effective antibiotics has necessitated the search for new alternative antimicrobial agents. Over the past decade, ribosomally synthesized natural peptides produced by a diverse group of bacterial species have received attention [1]. Compared to non-ribosomally synthesized antimicrobials, the ribosomally produced peptides are attractive for pharmaceutical applications as they could potentially be bioengineered to improve characteristics such as potency, stability, solubility etc. [2]. One group of compounds, namely the bacteriocins, have attracted great interest due to their high potency (often active in the nanomolar range) and heat stability [3].

It has been suggested that most bacteria produce at least one bacteriocin [4]. While the exact ecological function of bacteriocins is unknown, they may play a role in competition by directly killing competing bacteria, function as colonizing peptides or function as signalling molecules to communicate with other bacteria or the host [5]. Bacteriocin-producing bacteria have been isolated from a wide variety of sources including food [6], soil [7] and the intestines of fish and animals [8, 9]. In addition, several members of the human microbiota have been shown to produce bacteriocins. For example we have previously employed both functional and *in silico* approaches in our laboratory to identify novel bacteriocins from the human gut microbiome [10–15]. Skin-derived bacteria have also been shown to produce, or shown potential to produce, bacteriocins [16–21].

For the current study, we have focused on examining the antimicrobial ability of coagulase negative staphylococci (CoNS) that were isolated from human skin or human blood with the aim of identifying novel bacteriocin-producers. CoNS are considered part of the normal commensal bacteria of the skin and are thought to act as host guardians by targeting pathogens [22]. We postulate that bacteriocin production plays an important role in this function and in support of this hypothesis Nakatsuji and colleagues [23] have shown that antimicrobial-producing CoNS are deficient in subjects with atopic dermatitis and reintroduction of these strains decreased colonization with *Staphylococcus aureus*. While numerous lantibiotics have been identified from CoNS, including epidermin from *Staphylococcus epidermidis*, gallidermin from *Staphylococcus gallinarium* and hominicin from *Staphylococcus hominis* [24], analysis of the entire genome sequences of publicly available CoNS genomes suggests that there are potentially many novel CoNS-derived bacteriocins that have not yet been functionally characterized (M. Begley; unpublished data).

The aim of this study was to screen one hundred human-derived CoNS for antimicrobial activity using agar-based deferred antagonism assays with a range of indicator bacteria. One CoNS strain (CIT060) was selected for further investigation and it was identified as *Staphylococcus capitis* and herein we describe the purification and characterisation of the novel bacteriocin that the strain produces.

## Materials and methods

### Bacterial strains and growth conditions

The bacterial indicator strains used in this study are listed in Table 1. Strains were grown in either Brain Heart Infusion (BHI) broth at 37°C or in M17 broth supplemented with 0.5% glucose (GM17) at 30°C as indicated in the Table 1. All media was purchased from Oxoid and prepared according to the manufacturer’s recommendations.

**Table 1:** Bacterial strains used in this study.

Bacterial strains	Culture medium and temperature	Source
100 CoNS isolates	BHI at 37°C	CIT Culture Collection
*Staphylococcus epidermidis* TU3298 Positive control (epidermin producer), used during antimicrobial screening	BHI at 37°C	Teagasc, Culture Collection
*Staphylococcus capitis* CIT060	BHI at 37°C	CIT Culture Collection
**Indicator Strains**		
*Bacillus cereus* DPC 6089	BHI at 37°C	UCC culture Collection
*Enterococcus faecailis* MR103	BHI at 37°C	UCC culture Collection
*Geobacillus kaustophilus* DSM 7263	BHI at 55°C	UCC culture Collection
*Geobacillus stearothermophilus* ATCC 12930	BHI at 55°C	UCC culture Collection
*Lactococcus cremoris* IP5	GM17 at 30°C	UCC culture Collection
*Lactococcus lactis* HP	GM17 at 30°C	UCC culture Collection
*Lactococcus lactis* MG1363	GM17 at 30°C	UCC culture Collection
*Lactococcus lactis* NZ9800	GM17 at 30°C	UCC culture Collection
*Micrococcus luteus* DSM1790	BHI at 37°C	UCC culture Collection
*Staphylococcus aureus* 5971	BHI at 37°C	UCC culture Collection
*Staphylococcus aureus* DPC 5243	BHI at 37°C	UCC culture Collection
*Staphylococcus aureus* DPC5297	BHI at 37°C	UCC culture Collection
*Staphylococcus aureus* NCDO 1499	BHI at 37°C	UCC culture Collection
*Staphylococcus aureus* Newman	BHI at 37°C	UCC culture Collection
*Staphylococcus aureus* RF122	BHI at 37°C	UCC culture Collection
*Staphylococcus epidermidis* (UCC strain)	BHI at 37°C	UCC culture Collection
*Staphylococcus gallinarium* 4616	BHI at 37°C	UCC culture Collection
*Staphylococcus intermedius* DSM 20373	BHI at 37°C	UCC culture Collection
*Staphylococcus lugdunesis*	BHI at 37°C	UCC culture Collection
*Staphylococcus pseudintermedius* DSM 21284	BHI at 37°C	UCC culture Collection
*Streptococcus agalactiae* ATCC 13813	BHI at 37°C	UCC culture Collection
*Streptococcus dysgalactiae* ATCC43078	BHI at 37°C	UCC culture Collection
*Streptococcus pneumoniae* (UCC strain)	BHI at 37°C	UCC culture Collection
*Streptococcus pyogenes* NCDO 2381	BHI at 37°C	UCC culture Collection

BHI = Brain heart infusion; GM17 = M17 broth + 0.5% glucose; CIT = Cork Institute of Technology; UCC = University College Cork.

### Assembly of a bank of coagulase negative Staphylococci (CoNS)

The 100 CoNS strains were originally isolated from human skin swabs (obtained by swabbing the retro auricular crease i.e. behind the ear, the alar crease i.e. the side of the nose or the wrist), or from human blood samples at Cork University Hospital (CUH). Aliquots from archived stocks that are stored at -80°C at Cork Institute of Technology (CIT) were plated onto Mannitol Salt Agar (MSA) and incubated at 37°C for 16–18 hours. Single colonies were selected from the MSA plates and re-streaked onto BHI agar for purity determination. Strains were identified using Matrix-Assisted Laser Desorption/Ionization Time-Of-Flight (MALDI-TOF) at CUH. Briefly, single fresh colonies (from BHI agar plates incubated at 37°C for 16–18 hours) were directly applied to the MALDI-TOF stainless steel target plate. After application, each bacterial colony was covered with 0.8 μL of matrix solution (10 mg/mL α-cyano-4-hydroxycinnamic acid [HCAA] in 50% acetonitrile-2.5% trifluoroacetic acid) (Bruker Daltonik, GmbH, Germany). The data collected was classified in accordance to Bruker Taxonomy database of CUH.

The 100 strains were re-stocked in two separate master 96 well plates (50 in each plate). Overnight cultures of the 100 strains were prepared by selecting a single pure colony from BHI agar and adding it to 10ml of BHI broth and incubating at 37°C for 16–20 hours. After incubation, 100μl of each fresh overnight culture was added to a specific well in the 96 well plate, and 100μl of sterile 80% glycerol was added to each well. The master stock 96 well plates were stored at -80°C. Prior to use, the plates were thawed at room temperature.

### Screening of a bank of CoNS for antimicrobial activity

Agar-based deferred antagonism assays were carried out with a selection of indicator bacteria (listed in Table 1). The 100 CoNS strains were replicated onto BHI agar from the 96 well stock plates using a 96-pin replicator (Boekel). Plates were incubated at 37°C for 16–18 hours after which the surface of the agar plate was subjected to UV treatment for 30 minutes (High performance UV transmitter, Upland, Ca, USA). 30μL of fresh overnight cultures of indicator strains were added to 20ml of relevant sloppy/soft agar (BHI/GM17 broth supplemented with 0.75% w/v agar) and poured over the replicated plates. Plates were incubated at 37°C for 12–16 hours after which they were examined for zones of inhibition.

### Investigation of the antimicrobial activity of cell-free supernatants and crude whole cell extracts

Short-listed CoNS were grown overnight in BHI broth at 37°C under vigorous continued shaking (130 rpm), and a 1% inoculum of the cultures were added to 50 ml clarified BHI broth (prepared by passing BHI broth through XAD-16N beads (Sigma-Aldrich) prior to autoclaving) and incubated with vigorous continued shaking at 37°C. Following incubation, 50 ml of bacterial cells were centrifuged at 7,000 rpm for 20 minutes, supernatant removed and retained, i.e., cell-free supernatant (CFS). The cell pellets were resuspended in 7 ml 70% isopropanol (IPA) 0.1% trifluoroacetic acid (TFA) and stirred vigorously for 3 hours. Cell debris was removed through centrifugation and the supernatants were retained and referred to as whole cell extracts (WCE). CFS and WCE were examined for antimicrobial activity in an agar well diffusion assay using *L*. *lactis* HP as the indicator strain. 50ml of molten agar was seeded with 100 μL of *L*. *lactis* HP that was grown overnight at 30°C, and once solidified, 4.6mm holes were bored with a sterile glass pipette. 50 μL of CFS and WCE were added to separate wells. Plates were incubated at 30°C for 16–18 hours after which they were examined for zones of inhibition.

### Purification of capidermicin from *S*. *capitis* CIT060

Capidermicin was purified from *S*. *capitis* CIT060 using a method described by Field *et al*. [25] with modifications. Three litres of clarified BHI (cBHI) broth was inoculated (1%) with *S*. *capitis* CIT060 and incubated for 18–20 hours at 37°C under vigorous continued shaking (130 rpm). The culture was centrifuged at 7,000 rpm for 15 minutes. The cell pellet was removed, and the supernatant was retained and passed through 60g of Amberlite XAD16N beads (Sigma Aldrich). The beads were washed with 30% ethanol, and the peptide was eluted in 500ml 70% isopropanol (IPA) containing 0.1% trifluoroacetic acid (TFA). The IPA was evaporated using a rotary evaporator (Buchi) and the sample pH adjusted to 4 before applying to a 60 ml Strata C-18 E column (Phenomenex) that was previously pre-equilibrated with 60 ml methanol (Fisher Scientific, UK) and 60 ml H_2_O. 100 ml of 30% ethanol was used to wash the column and the peptide was eluted in 60 ml of 70% IPA, 0.1% TFA. 10 ml aliquots were concentrated to 2 ml through the removal of IPA by rotary evaporation. 2.0 ml aliquots were applied to a Phenomenex (Phenomenex, Cheshire, UK) C12 reverse phase (RP)-HPLC column (Jupiter 4u proteo 90 Å, 250 × 10.0 mm, 4 μm) previously equilibrated with 25% acetonitrile containing 0.1% TFA. The column was subsequently developed in a gradient of 25% acetonitrile containing 0.1% TFA to 50% acetonitrile containing 0.1% TFA from 10 to 45 minutes at a flow rate of 2ml min^-1^. The relevant active fractions were collected and pooled, subjected to rotary-evaporation to remove the acetonitrile and freeze-dried. The purity of the peptide was analysed by MALDI-TOF Mass Spectrometry [26].

### Minimum inhibitory concentration (MIC) assays

MIC determinations were carried out in triplicate in 96 well microtitre plates pre-treated with bovine serum albumin (BSA) as described by [27]. 200μL of 1X phosphate buffered saline (PBS) containing 1% (w/v) bovine serum albumin (PBS/BSA) was added to each well and incubated for 30 minutes at 37°C. The wells were washed with 200μL PBS and allowed to dry. Target strains were grown overnight in the appropriate medium and temperature conditions, sub-cultured into fresh broth and allowed to grow to an OD_600_ of approximately 0.5, and diluted to a final concentration of 10^5^ CFU/ml in a volume of 200μL broth. Lyophilised capidermicin was resuspended in cation adjusted BHI broth to a desired concentration, and a 2—fold dilution of the peptide was made in the 96 well plate. The target strain was then added and after incubation at 30°C or 37°C for 16 hours the MIC was read as the lowest peptide concentration causing inhibition of visible growth.

### Stability assays with capidermicin

The susceptibility of purified capidermicin peptide to temperature, pH and protease enzymes was investigated through well diffusion assays. To determine temperature stability the purified peptide was subjected to 10, 30, 40, 50, 80, 90, 121°C for 15 minutes. Bioactivity was then determined by carrying out an agar well diffusion assay with *L*. *lactis* HP as the bacterial indicator. To evaluate the susceptibility of the peptide to varying pH values, the purified peptide solution was adjusted to pH 2–11 using 1M HCl or 1M NaOH, respectively. After a brief vortex, the peptide was incubated at room temperature for 15 minutes, and the bioactivity was again determined with the well diffusion assay with *L*. *lactis* HP. The susceptibility of the peptide to proteolytic cleavage was analysed using trypsin, α-chymotrypsin, pepsin and proteinase K (Sigma-Aldrich). Protease enzymes were dissolved in 100 mM Tris-HCl– 10 mM CaCl_2_, to a final concentration of 100 μg/ml. Preparations of capidermicin were incubated with the various enzymes at 37°C for 4 hours and the bioactivity of capidermicin was reassessed using the well diffusion assay described above.

### Sequencing of *S*. *capitis* CIT060 genome

DNA extracted from *S*. *capitis* CIT060 was quantified using a Qubit high sensitivity assay (Invitrogen), and diluted to 0.2ng/μl. Genomic libraries were then prepared using the Nextera XT Library preparation kit (Illumina) essentially as described in the manufacturer’s protocol with the following exceptions. Firstly, the tagmentation time was extended to 7min. Following addition of indices, products were cleaned using AMPure XP magnetic bead-based purification, as described in the manufacturer’s protocol and then secondly, in place of the bead based normalisation, the products were run on an Agilent Bioanalyser to determine average fragment size (Agilent) and quantified again by Qubit. Cleaned genomic fragments were then pooled equimolarly. The sample pool (4nM) was denatured with 0.2N NaOH, then diluted to 6pM and combined with 10% (v/v) denatured 6pM PhiX, prepared following Illumina guidelines. Samples were sequenced on the MiSeq sequencing platform in the Teagasc sequencing facility, Moorepark, Fermoy, using a 2 x 300 cycle V3 kit, following standard Illumina sequencing protocols.

### *In silico* analyses of the predicted capidermicin gene cluster and encoded proteins

Following sequencing, the reads were assembled using Spades v. 3.5.0 [28]. Opening reading frames (ORFs) were predicted using Prodigal V.1.20 [29] and assigned a putative function based on BLASTp analysis at NCBI (http://www.ncbi.nlm.nih.gov/) and Pfam matches (EMBL-EBI) [30]. Any genomic regions which were identified as potentially containing antimicrobial-encoding genes were visualised using Snapgene Viewer (GSL Biotech; available at www.snapgene.com). These regions were manually annotated, and BLAST searches were performed with ORFs. Phyre2 (http://www.sbg.bio.ic.ac.uk/~phyre2) [31]) was used to generate a putative three-dimensional structure of the capidermicin peptide using two homologous peptides as templates—aureocin A53 (accession number AAN71834.1) and lacticin Q (accession number BAM66973.1). The sequence alignment of peptides of interest was performed using CLUSTAL OMEGA (https://www.ebi.ac.uk/Tools/msa/clustalo/).

## Results

### Screening a bank of CoNS strains for antibacterial activity

**Fig 1:**
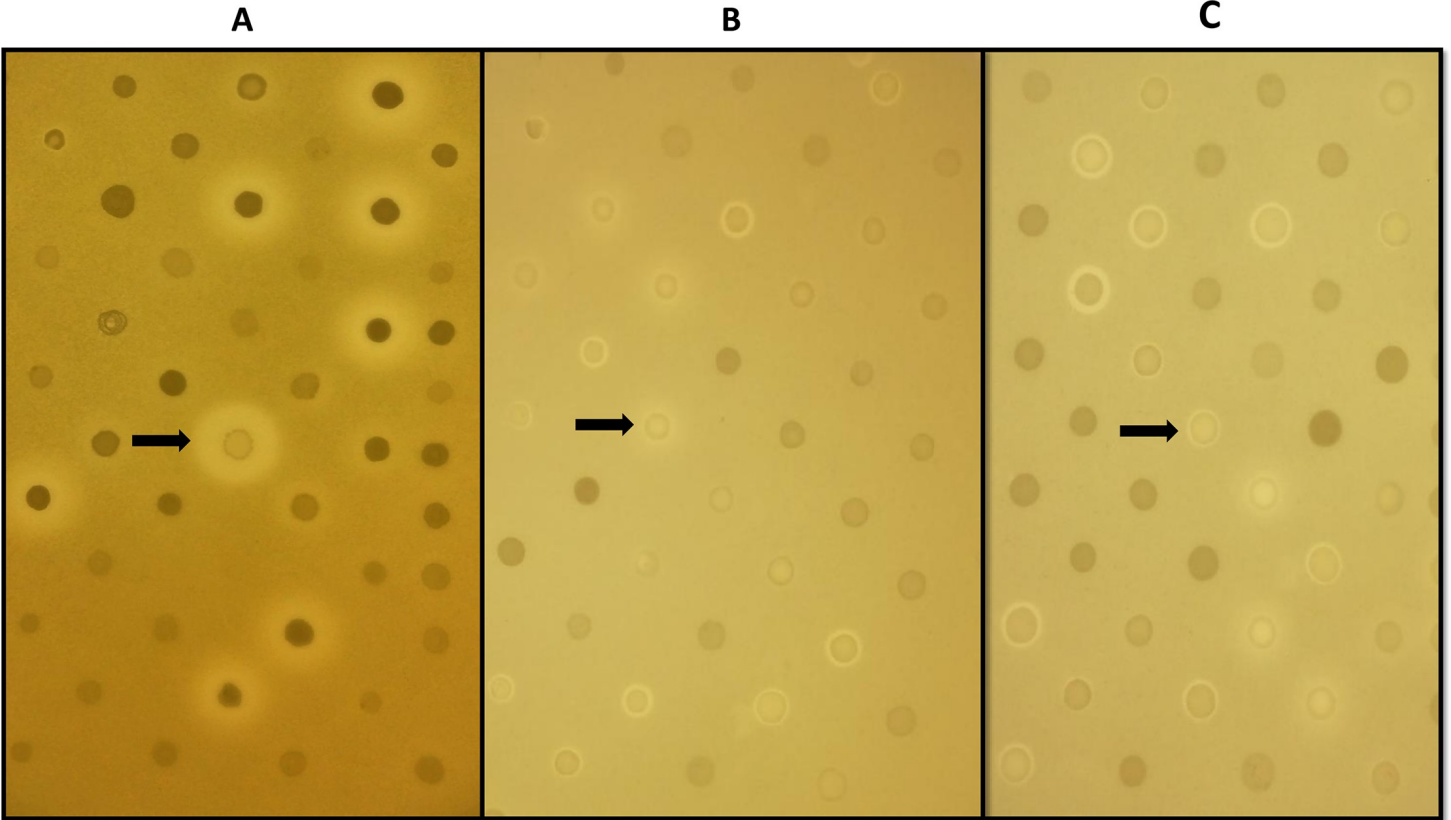
Representative images of the results obtained during the screen of 100 CoNS strains for antimicrobial activity. CoNS were replicated from master stock 96 well plates onto BHI agar using a 96-pin replicator. Plates were incubated at 37°C overnight after which they were overlaid with sloppy agar containing relevant indicator bacteria. For the plates shown the indicators used were (A) *M*. *luteus* DSM1790, (B) *S*. *aureus* NCDO1499 and (C) *S*. *pseudintermedius* DSM21284. The arrows indicate the position of *S*. *capitis* CIT060 on the plates.

A bank of 100 CoNS strains was assembled. All strains were phenotypically characterised using MSA agar plates and identified by MALDI-TOF analysis. The bank consisted of various staphylococcal species including 83 *S*. *epidermidis*, 7 *S*. *capitis*, 3 *S*. *haemolyticus*, 3 *S*. *hominis*, 2 *S*. *warneri*, 1 *S*. *saprophyticus* and 1 *S*. *simulans*. The 100 strains were examined using agar-based deferred antagonism assays for their ability to inhibit a selection of indicator strains (24 in total), including enterococci, lactococci, *Micrococcus*, streptococci and other *Staphylococcus* strains. Zones of inhibition were observed for 94 strains; representative images are shown in Fig 1. Six of the *S*. *epidermidis* strains did not demonstrate antimicrobial activity under the conditions tested. 15 of the 94 strains displayed a broad spectrum of activity, inhibiting 10 or more indicator strains. One strain, namely S. *capitis* CIT060, was capable of inhibiting 14 of the 24 bacterial indicators (*B*. *cereus* DPC6089, *E*. *faecailis* MR103, *G*. *kaustophilius* DSM7263, *L*. *lactis* subsp *cremonis* IP5, *L*. *lactis* HP, *M*. *luteus* DSM1790, *S*. *aureus* NCDO1499, DPC5297, Newman, and RF122, *S*. *lugdunensis*, *S*. *pseudintermedius DSM21284*, *S*. *intermedius* DSM 20373 and *S*. *dysgalactiae* ATCC43078).

### Purification of capidermicin from *S*. *capitis* CIT060

**Fig 2:**
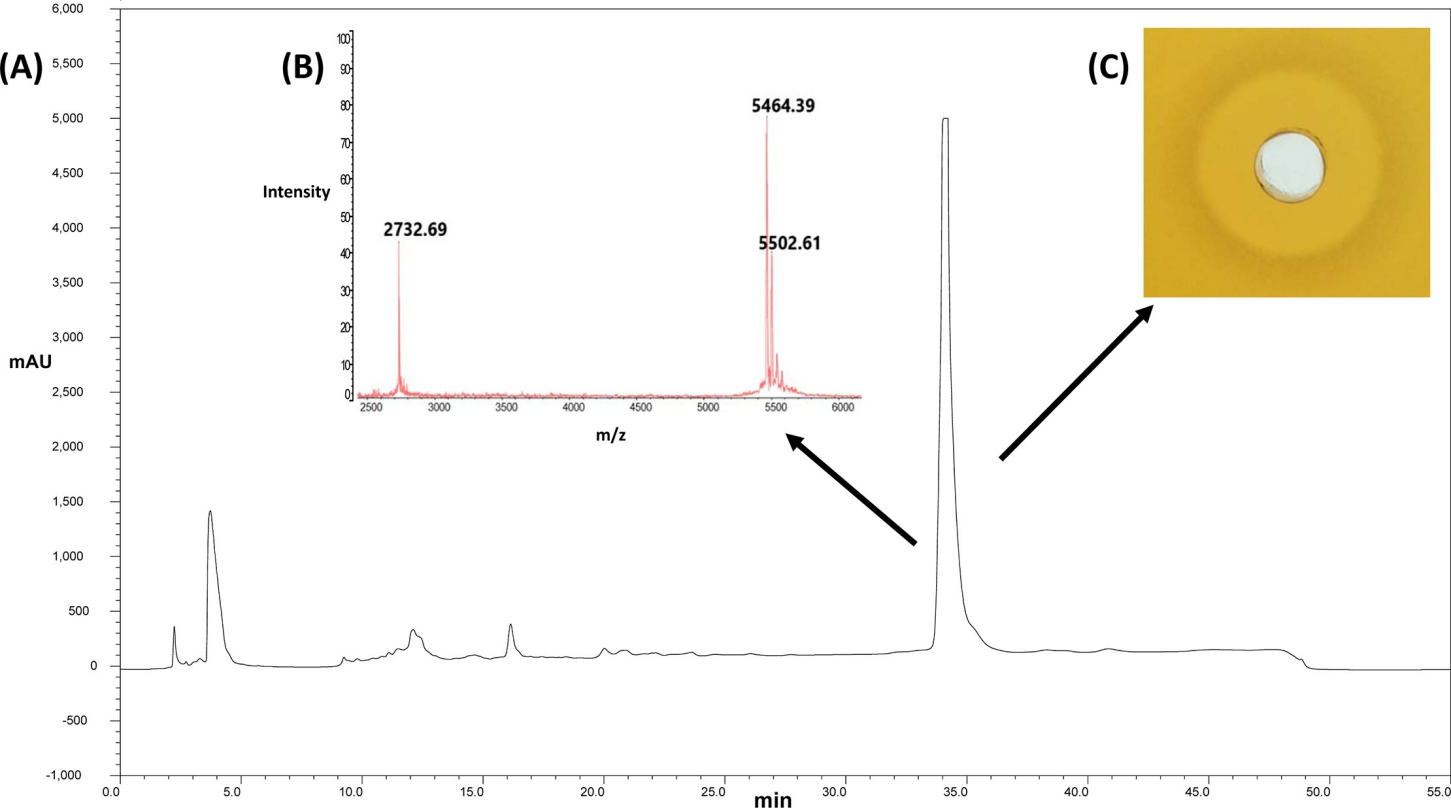
**(A)** Reversed–phase high performance liquid chromatography (RP-HPLC) profile for the purification of capidermicin using a Phenomenex C12 reverse-phase column, at a flow rate of 2ml/min. **(B)** MALDI-TOF Mass Spectrometry of lyophilized capidermicin revealed a mass of 5,464 Da. MALDI TOF MS chromatogram above indicates the presence of capidermicin (5464.39) and the K+ adduct ion (5502.61) and the doubly charged ion (2732.69) (2732 x 2 = 5464). **(C)** Antimicrobial activity of HPLC bioactive fractions was determined using well diffusion assay using *L*. *lactis* HP as the indicator strain. GM17 agar was seeded with *L*. *lactis* HP, wells were bored, 50μL of the HPLC fraction was added to the well, and plates were incubated at 30°C for 16 hours.

Initial experiments with cell-free supernatant and crude whole cell extracts prepared from *S*. *capitis* CIT060 suggested that the antimicrobial produced by the strain is primarily in the supernatant (data not shown). Consequently, efforts focused on purifying an antimicrobial peptide from culture supernatants as described in the Methods section. MALDI-TOF MS analysis revealed that the purified peptide, that we named capidermicin, had a mass of 5,464 Da (Fig 2).

The antimicrobial activity of purified capidermicin, as determined by agar well diffusion assays with *L*. *lactis* HP, remained unaffected following heat treatment at 10, 30, 40, 50, 80, 90 or 121°C for 15 minutes. The peptide was also able to maintain full bioactivity following exposure to pH 2–8. A 70% decrease in zone of inhibition observed at pH 9–11. Exposing capidermicin to α-chymotrypsin and pepsin had no effect on the bioactivity of the peptide. However, treatment with trypsin or proteinase K resulted in a 50% reduction in activity.

The specific activity of capidermicin was assessed using standard MIC broth-based assay and results are presented in Table 2. It was observed that capidermicin was active against the selected target bacteria in the nano- and micromolar range.

**Table 2:** Minimum inhibitory concentration (MIC) values of purified capidermicin against a range of Gram positive indicators. Identical MICs values were obtained in three independent determinations.

Species and Strain		
***Lactococcus lactis* HP**	19 μg/ml	3.4 μM
***Staphylococcus aureus NCDO 1499***	3.1 μg/ml	0.6 μM
***Staphylococcus aureus* SA113**	10 μg/ml	1.8 μM
***Staphylococcus intermedius* DSM20373**	40 μg/ml	7.3 μM
***Staphylococcus pseudintermedius* DSM21284**	10 μg/ml	1.8 μM
***Staphylococcus pseudintermedius* DK729**	10 μg/ml	1.8 μM
***Micrococcus luteus* DSM1790**	100μg/ml	18 μM

### *In silico* analyses of the predicted capidermicin gene cluster and encoded proteins

The draft genome of *S*. *capitis* CIT060 was analysed using a variety of *in silico* tools for the presence of potential antimicrobial peptide encoding genes. Three areas of interest were identified which included a lantibiotic gene cluster, a phenol soluble modulin (PSM) gene cluster and an aureocin-like gene cluster.

**Fig 3:**
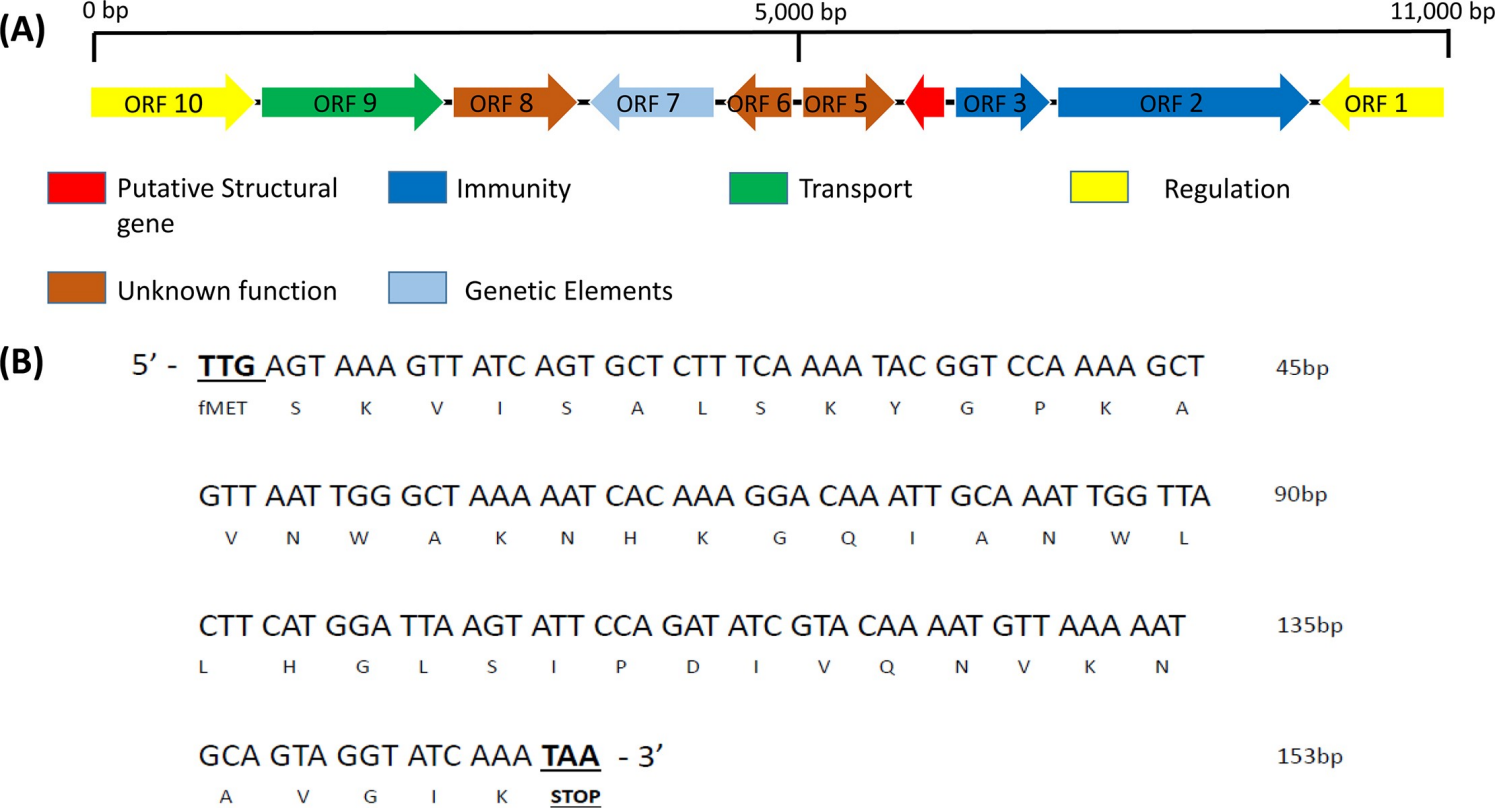
**(A)** Organisation of the genomic region that is predicted to encode capidermicin. Open reading frames (ORFs)/genes are coloured according to the predicted function. **(B)** The nucleotide sequence of the 153 bp ORF4 that is predicted to encode capidermicin. The deduced amino acid sequence is shown under the DNA sequence. The start and stop codon, TTG and TAA, respectively, are shown in bold and underlined.

**Fig 4:**
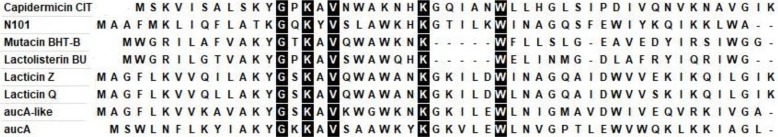
Alignment of the capidermicin amino acid sequence with homologous bacteriocins; epidermicin NI01 from *S*. *epidermidis* strain 224 (JQ025383), mutacin BHT-B from *Streptococcus ratti* BHT (DQ145753), lactolisterin BU from *L*. *lactis* subsp. *lactis* bv. diacetylactis BGBU1-4 (SDR48784), lacticin Q from *L*. *lactis* QU5 (BAF57910), lacticin Z from *L*. *lactis* QU14 (BAF75975), aureocin-like produced by numerous bacteria (SEM89646), and aureocin A53 from *S*. *aureus* A53 (WP_010976360). Highlighted residues indicate conserved amino acid sequences.

The first area of interest contains a gene that is predicted to encode a 44 amino acid peptide that is homologous to the lantibiotic gallidermin of S*taphylococcus gallinarum* (accession number U61158.1). The 44 amino acid peptide is predicted to encode a peptide with a mass of 5 kDa. However, a corresponding mass could not be detected from our bioactive HPLC fractions or purified capidermicin preparations. The second area of interest contains four genes that are predicted to encode PSMβ peptides. Phenol-soluble modulins (PSMs) are a recently discovered group of amphipathic peptides that have multiple roles in staphylococcal pathogenesis. They have been shown to exhibit antimicrobial activity [32]. All four predicted peptides contain the conserved domain of staph_haemo superfamilies (pfam05480), their amino acid sequences are identical to the PSMβ peptides previously reported in the literature [16], [21] and they are predicted to have masses of 4.57 kDa, 4.54 kDa, 4.62 kDa and 4.79 kDa. Again, corresponding masses could not be detected from our bioactive HPLC fractions or purified capidermicin preparations. The genetic organization of the final area of interest is shown in Fig 3A and the predicted functions of the putative gene products are shown in Table 3. *Orf4* was predicted to encode a peptide that is homologous to a number of previously characterised bacteriocins including lacticin Z produced by *Lactococcus lactis* QU14 (46% identity; accession number BAF75975), aureocin A53 produced by *Corynebacterium jeikeium* (41% identity; accession number WP010976360) and an aureocin-like bacteriocin produced by *Lactococcus ruminis* (57% identity; accession number SEM89646). More distant homologues include BacSp222 (32% identity; accession number A0A0P0C3P7) and Lactolisterin BU (44% identity; accession number SDR48784). A Clustal Omega alignment was carried out to compare the predicted peptide encoded by *orf4* and previously characterised bacteriocins, and the relatedness of the peptides is depicted in Fig 4.

**Table 3:** *In silico* analysis of the genes predicted to be involved in the biosynthesis of capidermicin.

					
**ORF 1**	60_02323	738	Plasmid replication initiator protein of *Listeria monocytogenes*	98% (WP_096929472.1)	Plasmid replication
**ORF 2**	60_02322	1407	YdbT-like protein of *S*. *aureus*	29% (WP_032072953.1)	Self-immunity
**ORF 3**	60_02321	405	YdbS-like protein of *S*. *intermedius*	38% (COG3402)	Self-immunity
**ORF 4**	60_02320	153	Aureocin A53 –like protein	46% (AF447813)	Structural gene
**ORF 5**	60_02319	558	Membrane protein of *Staphylococcus* sp.TE8	76% WP_082243241.1)	Unknown
**ORF 6**	60_02318	243	Hypothetical protein of *S*. *epidermidis*	95% (WP_049397332.1)	Unknown
**ORF 7**	60_02317	624	Transposase of *Staphylococcus*	100% (WP_017176851.1)	Transposon
**ORF 8**	60_02316	516	Hypothetical protein	99% (WP_020368224.1)	Unknown
**ORF 9**	60_02315	996	Putative sulfate exporter family transporter	100% (WP_070441690.1)	Transport of peptide
**ORF 10**	60_02314	822	Transcriptional Regulator	100% (WP_070441693.1)	Gene Regulation

The mass of the putative *orf4*-encoded peptide was predicted to be 5,438 Da by *in silico* tools (under Genbank accession MN234131). However, as the homologue aureocin A53 contains an N-formylated methionine [33]), and the start codon of *orf4* was noted to be TTG (Fig 3B), a revised theoretical mass of 5,466 Da was predicted. This mass is virtually identical to the mass of the capidermicin peptide that we purified from *S*. *capitis* CIT060 (Fig 2).

**Fig 5:**
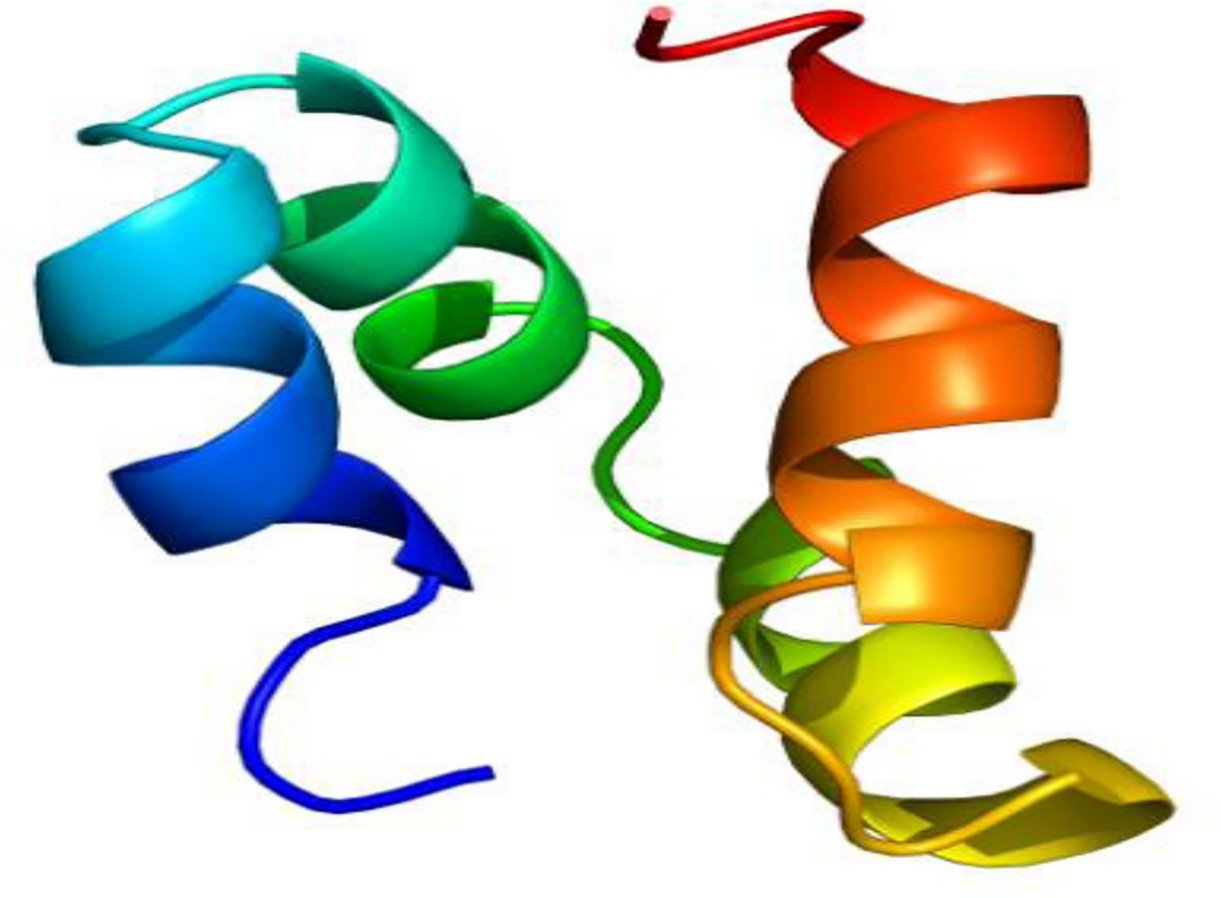
Putative three-dimensional structure of capidermicin. A rainbow colour scheme is used to indicate the N-terminus in blue, and the C-terminus in red. The structure was generated using Phyre2.

Capidermicin is predicted to be cationic with a putative net charge of 5.34 (theoretical pI = 10.22) and is rich in lysine residues (14%). A 3D structural model is presented in Fig 5. The peptide is predicted to be composed of four α-helices.

## Discussion

The aim of the current study was to screen 100 human-derived CoNS for antimicrobial activity. Agar-based deferred antagonism assays revealed that 94 of the strains reproducibly inhibited at least some of the tested indicator bacteria. Six strains did not show antimicrobial activity under the test conditions employed but it is possible that these strains may demonstrate activity under other conditions, such as different growth medium or different indicator bacteria. Janek and colleagues [18] carried out a similar agar-based screen to determine the frequency of antimicrobial production by 89 human nasal *Staphylococcus* isolates. 77 of the 89 isolates (86.5%) exhibited antimicrobial activity. When taken together, the findings of both studies suggest that antimicrobial production is a common phenotype among CoNS isolates. In contrast, a recent study by O’ Sullivan *et al*. [19], which describes a similar screen with human skin-derived staphylococci reports that only 101 possible antimicrobial-producers were identified from over 90,000 colonies that were screened for antimicrobial activity. The difference in the frequency of isolation of antimicrobial producers between that study (0.112%), the study by Janek *et al*. (86.5%) and our study (94%) may be because O’ Sullivan *et al*. used only one indicator organism in their screen (*Lactobacillus delbrueckii* subsp. *bulgaricus*) while a variety of indicators were used in the other two studies. It is possible that antimicrobials produced by CoNS may not inhibit *L*. *delbruecki* or activity may be too low to be detected in deferred antagonism assays.

Interestingly, functional screens of human intestine-derived bacteria report isolation of antimicrobial producers at a low frequency. Lakshminarayanan *et al*. [11] screened over 70,000 faecal bacteria for their ability to inhibit the indicators *Lactobacillus bulgaricus* LMG6901 and *Listeria innocua* DPC3572 and only identified 55 antimicrobial producers (a frequency of 0.08%). The *in silico*-based investigations of Zheng and colleagues [34] revealed that the human gut microbiome had the lowest frequency of putative bacteriocin genes of all the human body sites investigated. It is possible that production of antimicrobials by CoNS will increase their fitness in order to compete and survive on human skin. The limited availability of nutrients and water in this environment compared to the gastrointestinal tract may mean that the skin is a more competitive environment hence explaining the higher frequency of antimicrobial producers in screens with skin-derived bacteria compared to gut-derived bacteria. As humans and their microbes have co-evolved it is likely that production of antimicrobials by CoNS plays a beneficial role for the host, perhaps by contributing to the role of the skin as the body’s first line of defence by protecting against pathogenic bacteria. It is also possible that the antimicrobials may act as signalling molecules and interact with the human immune system [5].

The 94 CoNS that demonstrated antimicrobial activity in the initial screen were shortlisted to 15 based on their inhibition spectra and the activity of cell-free supernatants and whole cell extracts in well assays. *S*. *capitis* CIT060 was selected for further investigation as it demonstrated a broad inhibition spectrum and the results of well assays suggested that the antimicrobial could potentially be purified using methods that we currently use for bacteriocins in our lab [25]. We also noted that there are very few reports of functionally characterized *S*. *capitis* antimicrobials in the literature. While antimicrobial production by *S*. *capitis* strains has been shown by agar-based studies [18, 19] and antimicrobial genes have been identified in *S*. *capitis* genomes by *in silico* based methods [16, 35], to our knowledge the only two functional characterization studies in the literature are those of Sugai *et al*. [36], who reported the purification of the glycylglycine endopeptidase ALE-1 from *S*. *capitis* EPK1 that is similar to the bacteriocin lysostaphin, and Kumar *et al*. [21] who chemically synthesized and characterized phenol soluble modulins.

A 5,464 Da peptide, that we named capidermicin, was purified from *S*. *capitis* CIT060 cell free supernatants. The antimicrobial activity of the peptide was confirmed, and it was shown to retain its activity over a range of pH and temperatures. Analysis of the *S*. *capitis* CIT060 genome revealed the presence of 6 potential antimicrobial encoding genes (2 bacteriocins, 4 PSMs) but based on mass we deduced that the antimicrobial peptide that we purified was the product of the aureocin-like gene cluster. A gene encoding a potential 44 amino acid lantibiotic similar to gallidermin was also identified in the genome of *S*. *capitis* CIT060. Carson *et al*. [35] reported the presence of lanthipeptide gene clusters in the genomes of the 12 *S*. *capitis* isolates analysed (*S*. *capitis* 1319, 3379, 3769, 4275, 6079, 807, 1187, 1642, 2477, 2643, 4830 and 5871). Three of these strains were also shown to contain a sactipeptide gene cluster (*S*. *capitis* 1319, 2487 and 3379) [35]). Kumar *et al*. [21] identified four distinct gene clusters with the ability to encode antimicrobial peptides (epidermicin, gallidermin and phenol-soluble modulins) in *S*. *capitis* TE8. While the lantibiotic did not seem to be produced by *S*. *capitis* CIT060 under the experimental conditions used, the production of more than one bacteriocin by a bacterium is not uncommon. Lactococci commonly produce more than one bacteriocin and *Lactococcus lactis* subsp. *lactis* bv. diacetylactis BGBU1-4 strain produces at least two bacteriocins [37, 38]. Similarly, *Staphylococcus aureus* 4185, a bovine mastitis isolate, was shown to produce five antimicrobial peptides (named peptides A–E) [39].

*In silico* analyses suggest that capidermicin is a novel member of the class II leaderless bacteriocin family. It shows most similarity to members of the aureocin 53-like sub-group of the family which includes aureocin A53 produced by *S*. *aureus* A53 [33], lacticin Z produced by *L*. *lactis* QU14 [40], lacticin Q produced by *L*. *lactis* QU5 [41] and epidermicin NI01 produced by *S*. *epidermidis* strain 224 [42]. All of the bacteriocins within the group are 34–53 amino acid peptides, are highly cationic and are characterised by their lack of an N-terminal leader sequence during biosynthesis meaning that they do not undergo any post-translational modifications and become active shortly after translation [43]. While the genes encoding the immunity and secretion machinery have been experimentally determined for lacticin Q and Z [44], [45], aureocin A53 [46] and aureocin A70 [47]; [48], compared to other the classes of bacteriocins there is little known about the biosynthesis of leaderless bacteriocins and they have been referred to as the most enigmatic and poorly understood group of bacteriocins [43]. Similar to lacticin Q (48%) and aureocin A53 [49], capidermicin is predicted to be α-helical globular molecule.

MIC assays revealed that capidermicin was active against Gram-positive bacteria at low concentrations (μM/nM). Similar findings have been reported for epidermicin NI01 [42] and lacticin Q [41]. While capidermicin is insensitive to α-chymotrypsin and pepsin, a 70% reduction in activity was observed when it was treated with proteinase K or trypsin. A similar decrease in activity has previously been observed for epidermicin NI01, which displayed a 75% and 50% reduction in activity for proteinase K and trypsin, respectively [42]. Resistance to proteases has been reported for other staphylococcal bacteriocins, including aureocin A53 and BacCH91 [33]; [50]. Capidermicin showed high stability under acidic, alkaline and neutral conditions, which has also been reported for aureocin A53 [33], lacticin Z [40] and lacticin Q [41]). It has previously been noted that the high stability of leaderless bacteriocins together with the simplicity of their biosynthesis may make them more attractive from a commercial view point compared to other bacteriocins [43].

A 3D structural model is presented in Fig 5. The peptide is predicted to be composed of four α-helices, and exhibits a recurring three dimensional structural motif found among many linear leaderless bacteriocins (lacticin Q, aureocin A53) and similar to that found in a larger superfamily of proteins known as saposin-like peptides [51]. All the helices of capidermicin are amphipathic whereby hydrophobic residues are oriented inward that pack to give a hydrophobic core and the hydrophilic residues are exposed on the surface in the same manner as LnqQ (data not shown). Similarly, both peptides have highly cationic surfaces. Capidermicin has 7 lysine residues which are well distributed throughout its primary structure. Indeed, all lysine residues are situated on the surface and are in fact found in the exact same locations to LnqQ (in capidermicin K3, K10, K14, K23, K44 and K50). Notably, although LnqQ and the closely related aureocin A53 are composed of four distinct α-helical structures that are structurally identical to each other, both have varying antimicrobial activity spectrums against Gram-positive bacteria, [49]. Studies have shown that LnqQ permeates target membranes by forming toroidal pores (4.6–6.6 nm in diameter), which facilitate the leakage of cellular contents [52]. However, a more recent study demonstrated that LnqQ was able to induce cell death even without the formation of pores [53]. AucA was also proposed to permeabilize cell membranes causing the leakage of essential molecules, dissipation of membrane potential, and cessation of macromolecular synthesis but without the formation of discrete pores [54]. Given the structural similarity of capidermicin to LnqQ and A53, it is likely that capidermicin can also permeabilize cell membranes resulting in cell death.

In conclusion, we report that our screening experiment revealed a large frequency of antimicrobial production by human CoNS isolates and we describe the subsequent identification and characterization of a novel bacteriocin from *S*. *capitis*. Future work in our laboratory will include the chemical synthesis of the capidermicin peptide for additional *in vitro* experiments to confirm activity and antimicrobial spectrum, similar to studies conducted on other leaderless bacteriocins including garvicin KS and epidermicin NI01 [55, 56]. In the case of epidermicin NI01, chemical synthesis permitted the generation of sufficient peptide for further analyses including *in vivo* studies [57, 58]. Moreover, accessibility to capidermicin by chemical synthesis would provide a means for peptide engineering investigations to be carried out i.e. molecular engineering to enhance the potency, improve pharmacological properties, increase peptide stability and potentially modify the spectrum of activity. It may also provide more detail regarding the importance of the formylated methionine at the N-terminus for the antimicrobial activity of capidermicin. Future experiments will also include mutational analysis of all of the genes that are predicted to be involved in capidermicin production. A comprehensive gene disruption of the capidermicin biosynthetic cluster will be carried out, similar to that of Iwatani and colleagues [56] whereby each gene of the lacticin Q operon (lnqQBCDEF) was individually deleted and the impact on production and immunity evaluated.
